# *In vitro* uptake of apoptotic body mimicking phosphatidylserine-quantum dot micelles by monocytic cell line

**DOI:** 10.1186/1556-276X-9-176

**Published:** 2014-04-11

**Authors:** Andrei Maiseyeu, Vaishali Bagalkot

**Affiliations:** 1 Division of Cardiovascular Medicine, Department of Medicine, University of Maryland, Baltimore, 20 Penn St., HSFII, S012D, Baltimore, MD 21201, USA; 2 Department of Internal Medicine/Cardiology, University of Texas Health Science Center (UTHSC), 1881 East Road, South Campus Research Building 3, Center for Advanced Biomedical Imaging Research, 3SCR6.4610, Houston, TX 77054, USA

**Keywords:** Phosphatidylserine, Micelles, Quantum dots, Macrophages

## Abstract

A new quantum dot (QD) PEGylated micelle laced with phosphatidylserine (PS) for specific scavenger receptor-mediated uptake by macrophages is reported. The size and surface chemistry of PS-QD micelles were characterized by standard methods and the effects of their physicochemical properties on specific targeting and uptake were comprehensively studied in a monocytic cell line (J774A.1).

## Background

Macrophage plays an important role in the destabilization of atherosclerotic lesions. Molecular imaging approaches that target and image macrophages may be potentially useful towards predicting plaque vulnerability during the natural history of the disease [1-5]. Macrophages are effective efferocytes with the ability to recognize the externalized phosphatidylserine (PS) on the plasma membrane surface of apoptotic cells *via* the scavenger receptors and remove them from circulation and the arterial wall [6-9]. Phosphatidylserine is a naturally occurring phospholipid (PL) and its use for targeting macrophages may improve the biocompatibility of the contrast agent and avoid the use of exogenous targeting agents such as antibodies and peptides. This approach of using phosphatidylserine for targeting macrophages has been reported previously for magnetic resonance imaging of macrophage contents in atheroma with gadolinium-containing liposomes [10] but PS-containing micelles have not been reported. Lipid-polyethylene glycol (PEG) micelles have traditionally been used to solubilize hydrophobic drugs and solubilize hydrophobic nanoparticles into discrete clusters that can include either single or multiple nanoparticles in their cores and thus can achieve size tunability for particular application [11]. Micelles formed from lipid-PEG are biocompatible, non-toxic, and stable *in vivo *[11-16]. For the purpose of tracking the uptake of micelles by macrophages, QDs were incorporated into the micelle preparations because of its extreme brightness and photostability in real time imaging. Furthermore, QDs can be substituted by other inorganic nanoparticles such as gadolinium, iron oxide, gold, and tantalum for clinical translation. The PS micelles were further assembled with an amphiphilic polymeric surfactant, phospholipid conjugated to polyethylene glycol (PL-PEG) for the solubilization of hydrophobic nanoparticles (QD), improved dispersibility of micelles in physiological buffers and prolonged circulation *in vivo *[14]. However, PEGylation can potentially interfere with the interactions between ligand and cell surface receptor and reduce cellular uptake [17,18], a fine balance between stability and targeting for PEGylated nanoparticles were extensively studied. We hypothesize that the ratio of PL-PEG and PS shell coverage for 6- to 8-nm hydrophobic trioctylphosphine oxide (TOPO) quantum dot (QD) could be optimized for colloidal stability and targeting efficacy.

## Methods

### Materials

*L*-α-phosphatidylserine (PS), 1,2-distearoyl-sn-glycero-3-phosphoethanolamine-*N*-[methoxy (polyethylene glycol)-2000] (ammonium salt) (DSPE-mPEG, 2kDa) were purchased from Avanti Polar Lipids, Inc. (Alabaster, AL, USA). All other chemicals were obtained from Sigma-Aldrich Corporation (St. Louis, MO, USA). Dulbecco's modified Eagle's medium (DMEM), fetal bovine serum (FBS), phosphate-buffered saline (PBS), penicillin-streptomycin, and hydrophobic trioctylphosphine oxide (TOPO) QDs (QD 620nm) were purchased from Ocean Nanotech, Corp (Carlsbad, CA, USA). MTT assay kit was purchased from Roche Applied Science (Indianapolis, IN, USA). Lab-TekTM chamber slide system was purchased from Thermo Scientific/Nalgene Nunc International (Rochester, NY, USA). Vectashield mounting medium with DAPI was purchased from Vector Laboratories, Inc. (Burlingame, CA, USA). J774A.1 monocytic cell line was obtained from American Type Cell line Collection (ATCC) (ATCC® TIB67™). A 100-kD dialysis membrane was purchased from Spectrum Laboratories (Irvine, CA, USA).

### Preparation of PS-QD micelles

Micelles were prepared by the addition of hydrophobic QDs in chloroform to phospholipids (PLs) at each mole ratio (PEG/PS 100:0, 60:40, 50:50, 40:60, and 0:100) in hot water under vigorous stirring, followed by high-speed homogenization to form a uniform milky micro-emulsion. Unless otherwise mentioned, only PS mole ratio is shown and the remaining assumed for PL-PEG mole ratio (for example, PS (0) means micelles made entirely from phospholipid methoxy PEG, PS (40) means PS/PL-PEG mole ratio is 60:40). Briefly, the PLs at various mole ratios as indicated in Table 1 were first dissolved in water at 50°C and QD 620 (0.2 nmol) dissolved in chloroform was added to PLs in water and briefly sonicated for a few minutes. Next, the emulsion of lipids and QDs were thoroughly mixed by a high-speed homogenizer and maintained in a hot water bath at 70°C under vigorous stirring inside a hood to evaporate the chloroform solvent, resulting in the solubilization of hydrophobic QDs in water. The obtained PS-QD micellar suspension was further purified to remove excess PLs by overnight dialysis against phosphate buffer (PBS) saline using a 100-kD dialysis cutoff membrane.

**Table 1:** Preparation and physico-chemical characteristics of PS-QD micelles

	**Polar lipids (mg)**	**PS (mg)**	**QD (620 nm; 2-μM concentration)**	**Clarity of emulsion**	**Stability of flourescence**	**Average size (by intensity; in nm)**	**Polydispersity index (PDI)**	**Zeta potential charge (in mV)**
QD-PEG-PS mole ratio	DSPE-PEG (2000) methoxy							
100:0, PS (0)	4.5	-	0.2 nmol	Clear	Quenched after 45 days	198.3	0.24	-8.7
60:40, PS (40)	2.7	1.8	0.2 nmol	Clear	Stable	104.6	0.18	-16.4
50:50, PS (50)	2.25	2.25	0.2 nmol	Clear	Stable	40.9	0.14	-14.5
40:60, PS (60)	1.8	2.7	0.2 nmol	Hazy	Stable	143.0	0.16	-21.8
0:100, PS (100)	-	4.5	0.2 nmol	Hazy	Stable	127.3	0.22	-32.2
QD-PEG-COOH	DSPE-PEG (2000) carboxylic acid							
	4.5	-	0.2 nmol	Clear	Stable	60.1	0.22	-25.3

### Physico-chemical characterization of PS-QD micelles

The mean hydrodynamic diameter, polydispersity index and zeta potential charge of PS-QD micelles was measured using a Zeta Nanosizer ZS (Malvern Instruments Ltd, Worcestershire, UK; Table 1). For size measurements, the PS-QD micelles were diluted (1:100) in 100-mM PBS buffer and for zeta potential measurements the PS-QD micelles were diluted (1:1,000) in 10-mM PBS buffer. All samples were measured in triplicate. The morphology of PS-QD micelles was analyzed by transmission electron microscopy (TEM; JEM1010; JEOL, Tokyo, Japan) operating at 60kV. For the preparation of PS-QD micelles for TEM, PS-QD micelles were diluted in distilled water and dropped on Formvar-coated copper grids. Samples were examined with and without negatively staining with osmium tetroxide.

### *In vitro* stability of PS-QD micelles

The colloidal stability of PS-QD micelles was analyzed by incubating PS-QD micelles in cell culture medium containing 10% fetal bovine serum (FBS). Four-hundred microliters of PS-QD micelles (QD concentration 1 μM) were diluted in 800 μL of cell culture media and placed in a 37°C water bath for 24 h. After 24 h, 0.5 mL of the micelle solution in media was diluted twice with PBS buffer (0.1M) for particle size analysis using a Zeta Nanosizer ZS.

### *In vitro* cell uptake (fluorescence microscopy and flow cytometry studies)

The cellular uptake and distribution of PS-QD micelles were semiquantitated by fluorescence microscopy and flow cytometry. After the J774A.1 cells reached 80% confluency, the cells were detached by a scraper and seeded onto a 6-well plate at a density of 2 × 10^4^ cells per well and incubated overnight. The culture medium was removed and PS-QD micelles PS (0), (40), (50), (60), and (100) at 10-nM concentration were added and incubated for 4h at 37°C. After incubation, the solution was removed and the cells were washed with PBS for at least three times. After washing with PBS, cells were scraped and centrifuged, the supernatant was carefully removed. PBS buffer containing 2% (*v*/*v*) FBS was added to the cell pellet and resuspended. The cells were analyzed using a FACS Calibur fluorescence-activated cell sorter (FACS™) equipped with Cell Quest software (Becton Dickinson Biosciences, San Jose, CA, USA). For flourescence microscopy, J774A.1 cells were seeded onto 4-well chamber slides at a density of 4.0 × 10^3^ per well (surface area of 1.7cm^2^ per well, 4-chamber slides) and incubated for 24h at 37°C. The PS-QD micelles PS (0), (40), (50), (60), and (100) at 10-nM concentration were added to the cells and incubated for 4h at 37°C. After incubation, the solution was removed and the cells were washed with PBS for at least three times. The cells were fixed with 4% formalin for 10min and washed with PBS and mounted with the DAPI mounting medium for nuclear staining. The cells were examined by an epifluorescence microscope (NIKON Eclipse) using separate filters for nuclei, DAPI filter (blue), and for QD (620); TRITC filter (red).

### Cell cytotoxicity

J774A.1 macrophage cells were cultured with DMEM supplemented with 10% FBS, 100 U/mL penicillin, and 100μg/mL streptomycin in a 5% CO_2_ atmosphere at 37°C. The cytotoxicity of PS-QD micelles on J774A.1 cells was evaluated using a colorimetric MTT assay kit. After the cells achieved 80% confluency, the cells were scraped and seeded onto a 96-well plate at a density of 1.5 × 10^4^ cells per well. After 24h of incubation, the cell culture medium was removed. All PS-QD micelles were filtered using a 0.45-μM syringe filter before addition to the cell culture medium. PS-QD micelles PS (0), (40), (50), (60), and (100) at concentrations of 1-, 5-, 10-, and 50-nM concentrations were incubated with the cells for 24 h at 37°C under a 5% CO_2_ atmosphere. After incubation, the medium was removed and the cells were washed with PBS three times. Fresh medium was added to the wells with 10 μL of MTT reagent at 37°C for 4 h according to the manufacturer's protocol. The absorbance was read at a wavelength of 550 nm with a spectramax microplate reader (Molecular Devices, Sunnyvale, CA, USA). The assay was run in triplicates.

## Results and discussion

**Figure 1:**
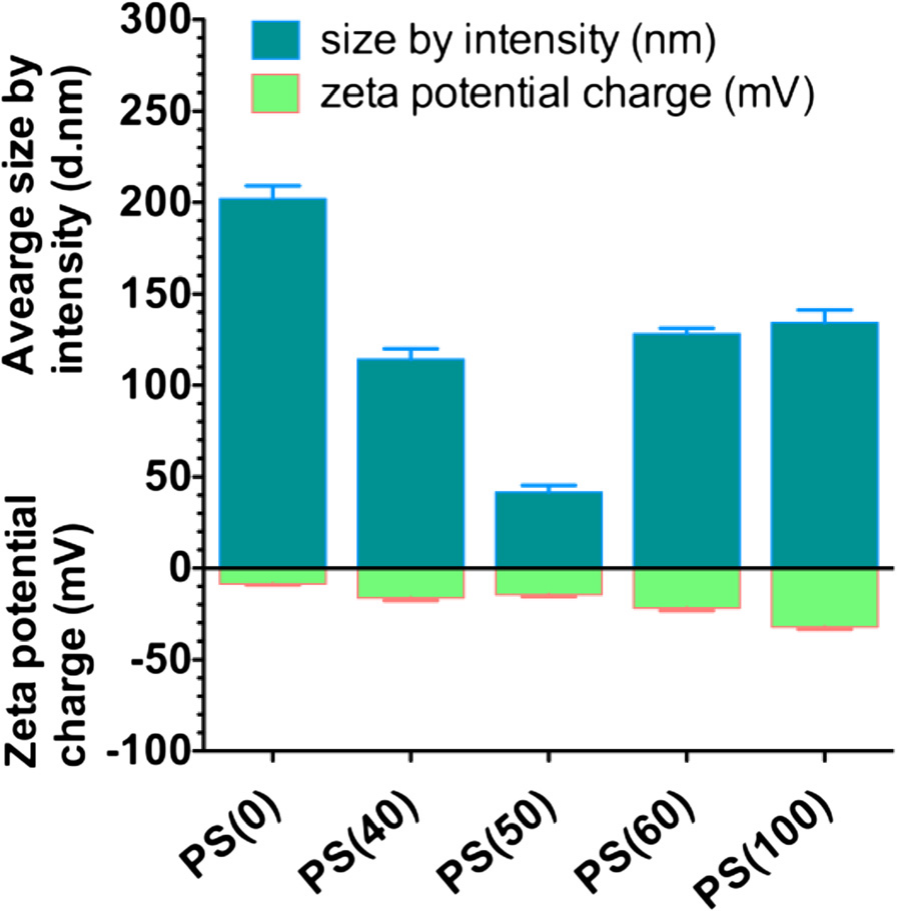
**Physico-chemical characterization of PS-QD micelles by dynamic light scattering.** The mean hydrodynamic diameters of micelles with varying PL-PEG/PS mole ratio. PS (0, 40, 50, 60, 100) micelles were 198.3 ± 3.7, 104.6 ± 9.7, 40.9 ± 0.5, 133.6 ± 17.9, and 127.3 ± 23.3 nm, respectively. The zeta potential values were -14.5 ± 7.5mV for PS (50) micelles, -16.4 ± 6.9mV for PS (60) micelles, to -32.5 ± 7.8mV for PS (100) micelles, respectively.

The molecular self assembly of QDs and PLs was accomplished by the addition of hydrophobic QDs to PLs in an organic solvent in hot water under vigorous stirring, followed by high-speed homogenization to form a uniform milky micro-emulsion. After the evaporation of the organic solvent at 40°C to 60°C for about 10 min, micellar PS-QD nanoparticles are formed (Table 1, Additional file 1: Figure S1). The micellar PS-QD nanoparticles were characterized by dynamic light scattering (DLS) and zeta potential measurements (Table 1). Unless otherwise mentioned, all mole ratios represent the molar ratio of PS/PL-PEG (methoxy) and only PS is denoted for clarity. The size distribution of QD-micelles formed entirely with PL-PEG (PS (0)) were 198.3 ± 3.7 nm (Figure 1, Additional file 1: Figure S3). Up to 50 mol% occupancy of PEG, the results are consistent with prior reports demonstrating the linear relationship between the hydrodynamic diameter of nanoparticles and PEG density [19]. However, with further decrease in PL-PEG, the size of PS micelles increased. The mean hydrodynamic diameter of PS (60) micelles was 133.6 ± 17.9 nm and that of PS (100) micelles with no PEG was 127.3 ± 23.3 nm. Transmission electron microscopy (TEM) was performed to further characterize the morphology of the PS (50) micelles. Negatively stained PS (50) micelles appear as small unilamellar vesicular structures with a size of approximately 50 nm with about 2 to 3 QDs seen within each micelle (Additional file 1: Figure S2). With increasing PS, the surface charge of PS-QD micelles increased from -14.5 ± 7.5 mV for PS (50) micelles, -16.4 ± 6.9 mV for PS (60) micelles, to -32.5 ± 7.8 mV for PS (100) micelles (Figure 1). Another important consideration when preparing nanoparticles for *in vivo* use is their colloidal stability in serum. The aggregation property of the micelles was studied by monitoring the change in their hydrodynamic diameter after 24 h of incubation with 10% (*v*/*v*) serum-containing media. The stability of PS-QD micelles decreases with increasing concentration of PS, PS (40) > PS (50) > PS (60) > PS (100) (Additional file 1: Figure S4). The results suggest that an amount of 50 to 60 mol% PEG for PS-PL-PEG micelles with 6- to 8-nm hydrophobic QD core is optimal for generating uniformly small micelles, for further evaluation. *In vitro* cytotoxicity of various PS-QD micelle preparations was also evaluated in J774A.1 cells. Up to 50 nM, all preparations of PS-QD micelles were found to be non-toxic to macrophages when incubated for 24 h, as assessed by MTT cell viability assay (Additional file 1: Figure S7).

**Figure 2:**
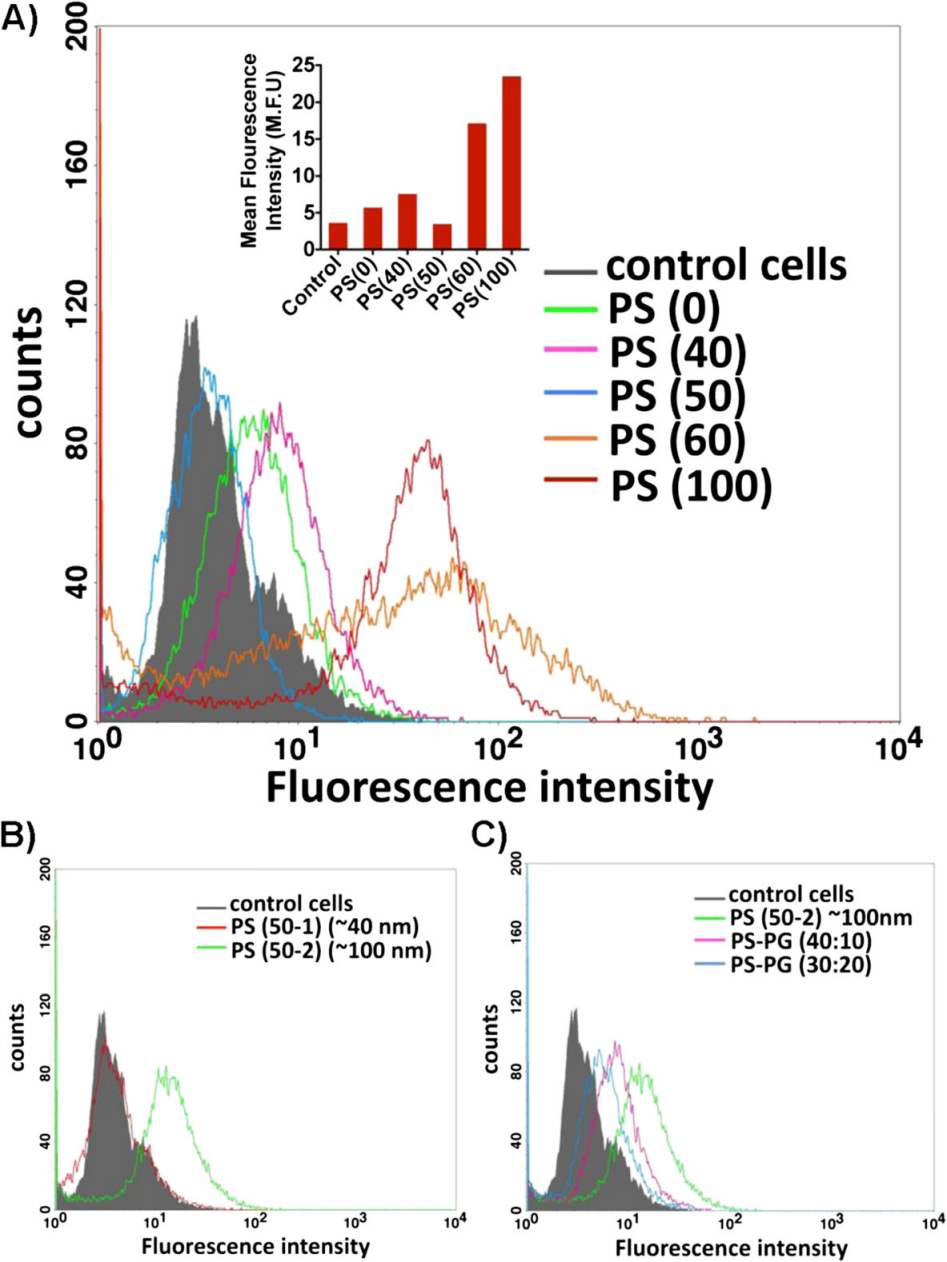
**Flow cytometry histogram profiles of untreated control cells (gray colored) versus PS-QD micelle-treated macrophage cells. (A)** Varying PS-QD micelles: PS (0, 40, 50, 60, and 100) incubated with macrophages for 4 h and analysed by flow cytometry, inset shows mean flourescence intensity values of the histograms, **(B)** histogram profile of macrophage cells treated with PS (50-1) and PS (50-2) micelle formulation, **(C)** histogram profile of macrophage cells treated with PS (50-2) QD micelle showing PS-dependent macrophage uptake.

**Figure 3:**
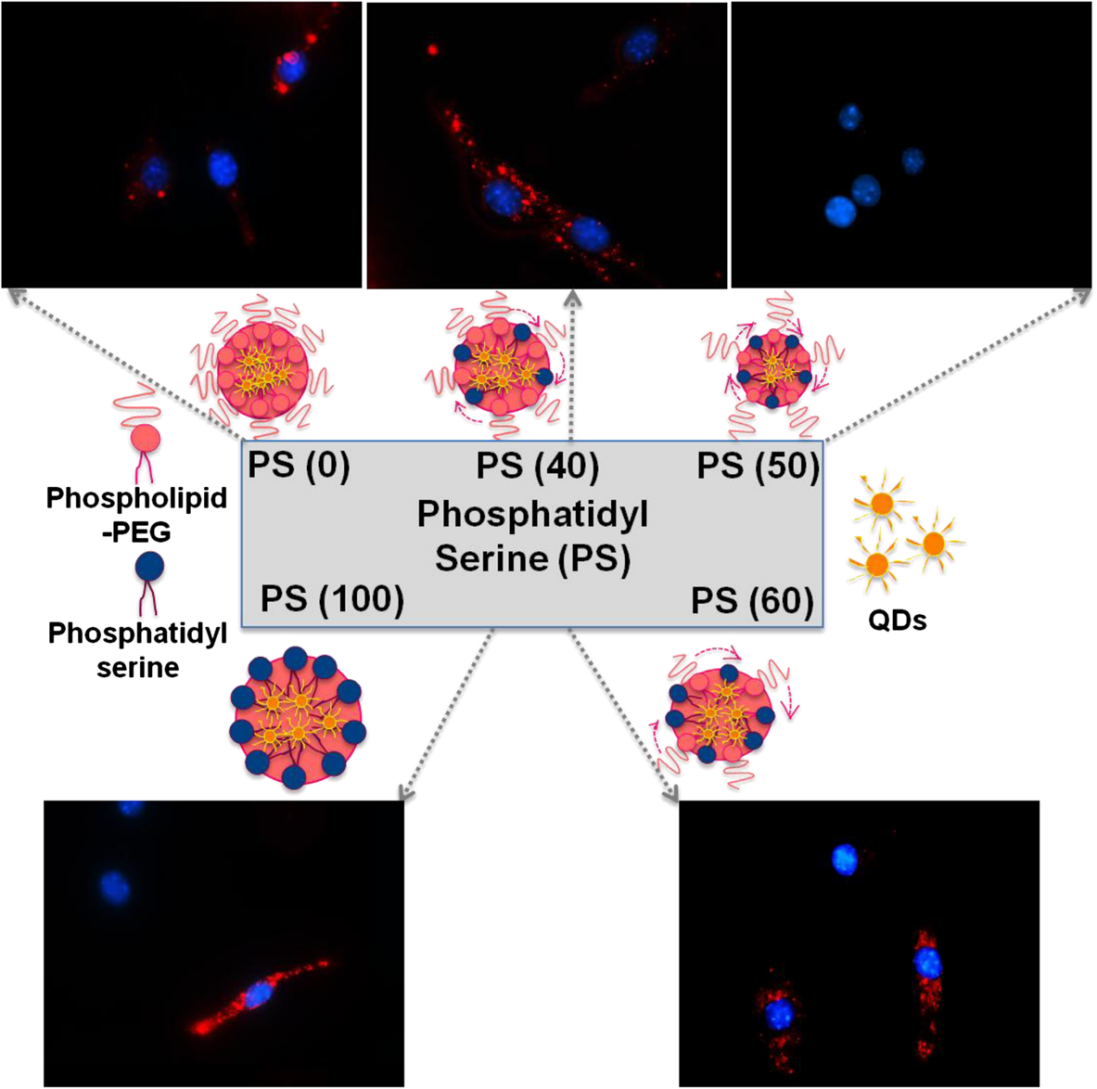
**Schematic representation of PS-QD micelles and evaluation of their targeting efficacy.** Uptake of PS-QD micelles by J774A.1 macrophages was tested as a function of micelle size and PS coverage. The uptake was highest for PS (100) and minimal for PS (50).

To demonstrate the ability of PS-QD micelles to target and subsequently phagocytosed by macrophages, J774A.1 cells were incubated with PS-QD micelles containing variable amount of PS (40, 50, 60, and 100 mol% PS). The extent of micelle uptake by macrophages was quantified by fluorescence-activated cell sorting (FACS). It was hypothesized that increasing PS mol% and decreasing PL-PEG packing density on micelles would determine the rate of internalization of PS-QD micelles by macrophages. The principles of protein interactions and PEGylated surfaces are well known and states that PEG adopts different confirmations at high and low packing densities [20]. As expected, the uptake of PS micelles by macrophages increased with increasing PS mol% (Figure 2, Additional file 1: Figure S5-S6) with the exception of PS (50) micelles. PS micelles with low PEG and high PS content: (i) PS (100) micelle treated macrophages showed nearly fourfold increase in cell uptake compared to PS (0) micelles and the cell count (histogram peak height) was similar to (histogram peak height) control untreated cells, demonstrating that all cells take up PS (100) micelles (mean fluorescence intensity (MFI) 23.4 versus 5.6), even though they form 2-μm particles when incubated in culture media, this result indicates that micron-sized particles are uptaken by macrophages. (ii) PS (60) micelles showed a threefold increase in cell uptake (MFI 17 versus 5.6) but the cell count (histogram peak height) was half that of PS (0) treated macrophages indicating that not all the micelles are internalized by macrophages resulting in lower number of cells containing PS-QD micelles (Figure 2A). For PS micelles with high PEG and low PS content, (iii) the uptake of PS (0) micelle by macrophages was not significant compared to untreated control (MFI 5.6 versus 3.5), (iv) PS (40) with a mean particle size of approximately 80 to 100 nm, showed only a onefold increase in cell uptake compared to PS (0) micelles (MFI 7.4 versus 5.6), and (v) PS (50) micelles (approximately 40 nm) showed no cell uptake, almost no change in QD peak intensity and were similar to control untreated cells (MFI 3.3 versus 3.5; Figure 2A). The results demonstrate that high PEG density on micelles results in closely packed PEG surface that resembles a brush type conformation, resulting in blocking PS recognition by macrophages [20,21]. Consistent with prior reports that demonstrated PEGylation on the surface of QD could substantially block the uptake of 15- to 30-nm particles by macrophages [19], the PS (50) micelles with 50 mol% PEG appeared to evade uptake by J774A.1 cells as assessed by flow cytometry (Figure 2). Fluorescent microscopy also confirmed the lack of uptake of PS (50) micelles by J774A.1 cells (Figure 3). It has been reported that PEG density affects macrophage uptake more for smaller sized nanoparticles compared to larger nanoparticles [19] and the results are in agreement. We therefore hypothesized that by increasing the micelle size, a fine balance between colloidal stability and macrophage targeting can be achieved.

Next, the PEG packing density of PS (50) micelles was controlled by tuning the homogenization speed of the micro-emulsion that resulted in the preparation of micelles of two different sizes of approximately 40-nm PS (50-1) and approximately 100-nm PS (50-2) micelles. When tested for macrophage-specific targeting, it was found that PS (50-1) micelles with a size of approximately 40 nm were not uptaken by macrophages (incubated at 25 pM) and at different micelle concentrations (Additional file 1: Figure S6), while PS (50-2) micelles with a size of approximately 100 nm in size are avidly uptaken by macrophages (MFI 15.1 versus 5.6) (Figure 2B). Further, the possibility that the uptake of larger-sized PS (50-2) micelles by macrophages were indeed correlated to the surface coverage of PS in the micelles and independent of surface negative charge was also investigated. For this purpose, the amount of PS in the PS (50-2) micelles was varied by substituting PS with a negatively charged lipid: 1,2-dipalmitoyl-sn-glycero-3-phospho-(glycerol) (DPPG) at two PS-DPPG molar ratios (40:10 and 30:20) but keeping the overall molar ratio constant at 50 mol%). As shown in Figure 2C, PS-PG (40:10) micelles containing more PS than PS-PG (30:20) micelles were taken up to a higher degree by macrophages, suggesting macrophage uptake of micelles was dependent on the PS content in micelles and independent of the surface charge. The above results show that PEG coverage and size can be fine-tuned to influence the surface exposure of PS and thus permit or block the ligand receptor recognition and cell uptake.

## Conclusions

In conclusion, a size-dependent uptake of approximately 100-nm PS-QD micelles that resemble dead/apoptotic cells and recognized as ‘self’ are detected and uptaken by macrophage-like cells, whereas PS-QD micelles that are intermediate in size (approximately 40 nm) and recognized as ‘non-self’ are not uptaken by macrophage-like cells. The importance of this study based on the size and phospholipid coating of equal molar ratio of PS and PL-PEG for nanoparticles can be further extended to targeted delivery of inorganic particles for imaging or drug delivery applications.

## Competing interests

The authors declare that they have no competing interests.

## Authors’ contributions

VB carried out the synthesis of PS-QD micelles, cell uptake studies and drafted the manuscript, AM edited and prepared manuscript for publication. All authors read and approved the final manuscript.

## Supplementary Material

Additional file 1: Figure S1-S7 Supporting Information. *In vitro* uptake of apoptotic body mimicking phosphatidylserine-quantum dot micelles by monocytic cell line. Click here for file
