# Supplementary material for: In vitro uptake of apoptotic body mimicking phosphatidylserine-quantum dot micelles by monocytic cell line
Source: Nanoscale Res Lett. 2014 Apr 11;9(1):176. doi: 10.1186/1556-276X-9-176 (PMC4022335; doi:10.1186/1556-276X-9-176)
Supplement: Additional file 1: Figure S1-S7 — Supporting Information. In vitro uptake of apoptotic body mimicking phosphatidylserine-quantum dot micelles by monocytic cell line. [file 1556-276X-9-176-S1.docx]

Supporting Information

*In vitro* uptake of apoptotic body mimicking phosphatidylserine-quantum dot micelles by monocytic cell line.

Vaishali Bagalkot*

*Department of Internal Medicine/Cardiology, University of Texas Health Science Center (UTHSC), 1881 East Road, South Campus Research Building 3, Center for Advanced Biomedical Imaging Research, 3SCR6.4610, Houston, Texas, 77054, USA.*

*E-mail:* [*Vaishali.bagalkot@uth.tmc.edu*](mailto:Vaishali.bagalkot@uth.tmc.edu)

**Corresponding Author: Vaishali Bagalkot, Ph.D.*


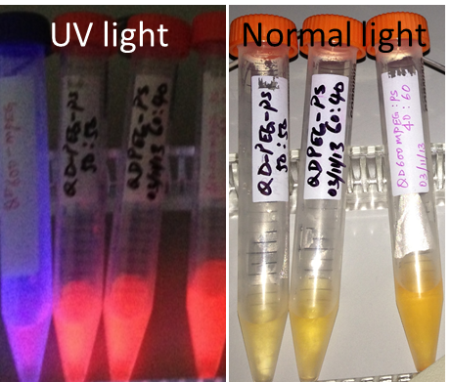


Fig. S1. Representative photograph of PS-QD micelles as seen under a hand held UV lamp (short wave length) (left) and ambient light (right).


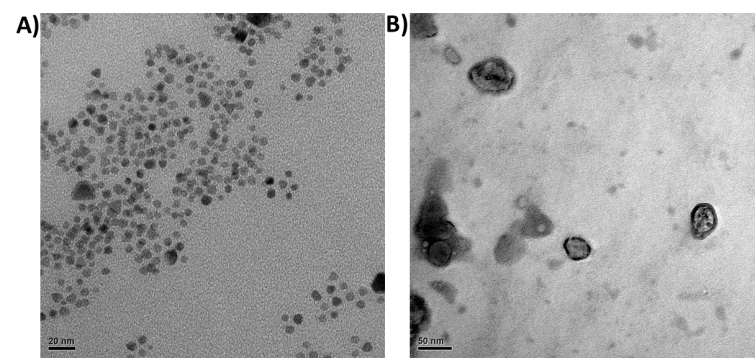


Fig. S2. TEM image of PS (50) micelles. (A) PS (50) micelles without negative staining. The dark spheres represent QD core nanoparticles with an size range of 8-10 nm (scale bar = 20 nm) and (B) PS (50) micelles negatively stained with osmium tetroxide. Negative staining demonstrates the unilamellar phospholipid shell of the micelles with an approximate size of 50 nm. The white arrow indicates the encapsulation of QDs inside the phospholipid-coated micelles (Scale bar = 50 nm).

**
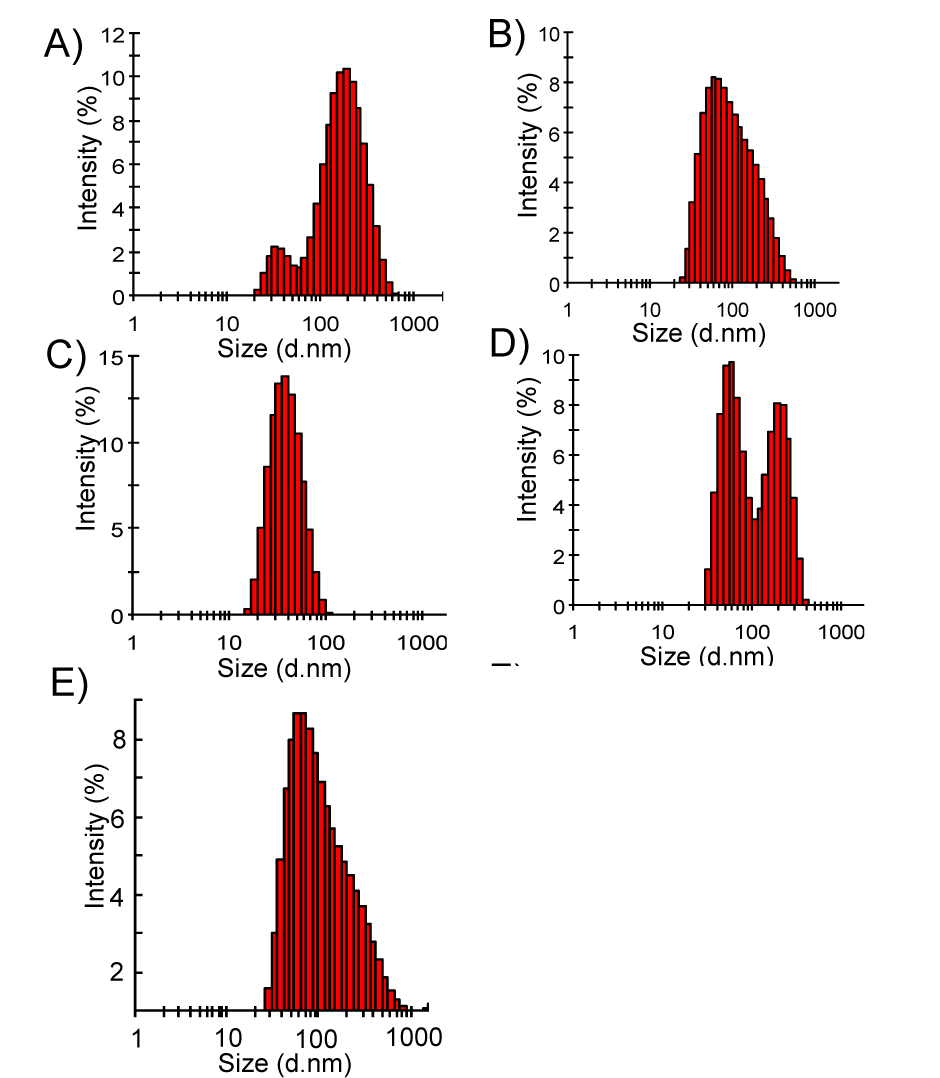
**

Fig. S3. Physico-chemical characterization of PS-QD micelles by dynamic light scattering. Size distribution analysis based on intensity distribution is shown. The PS-QD micelles with varying PL-PEG:PS mole ratio were diluted in 0.1M PBS buffer and their size distribution were measured by a Zeta Nanosizer ZS (Malvern Instruments Ltd, Worcestershire, UK). (A) PS (0) showed bimodal size distribution, minor peak at 38.5 ± 10.7 nm and major peak at 200 ± 94.9 nm, (B) PS (40) had an average diameter of 115.4 ± 83.8 nm, (C) PS (50) had an average diameter of 40.5 ± 15.5 nm, (D) PS (60) showed bimodal size distribution, peak 1 at 62.0 ± 19.0 nm and peak 2 at 200 ± 62.4 nm, (E) PS (100) had an average diameter of 133 ± 100.7 nm (± indicates width of the peak intensity) respectively, three replicates of each sample was analyzed by DLS with three runs per sample.


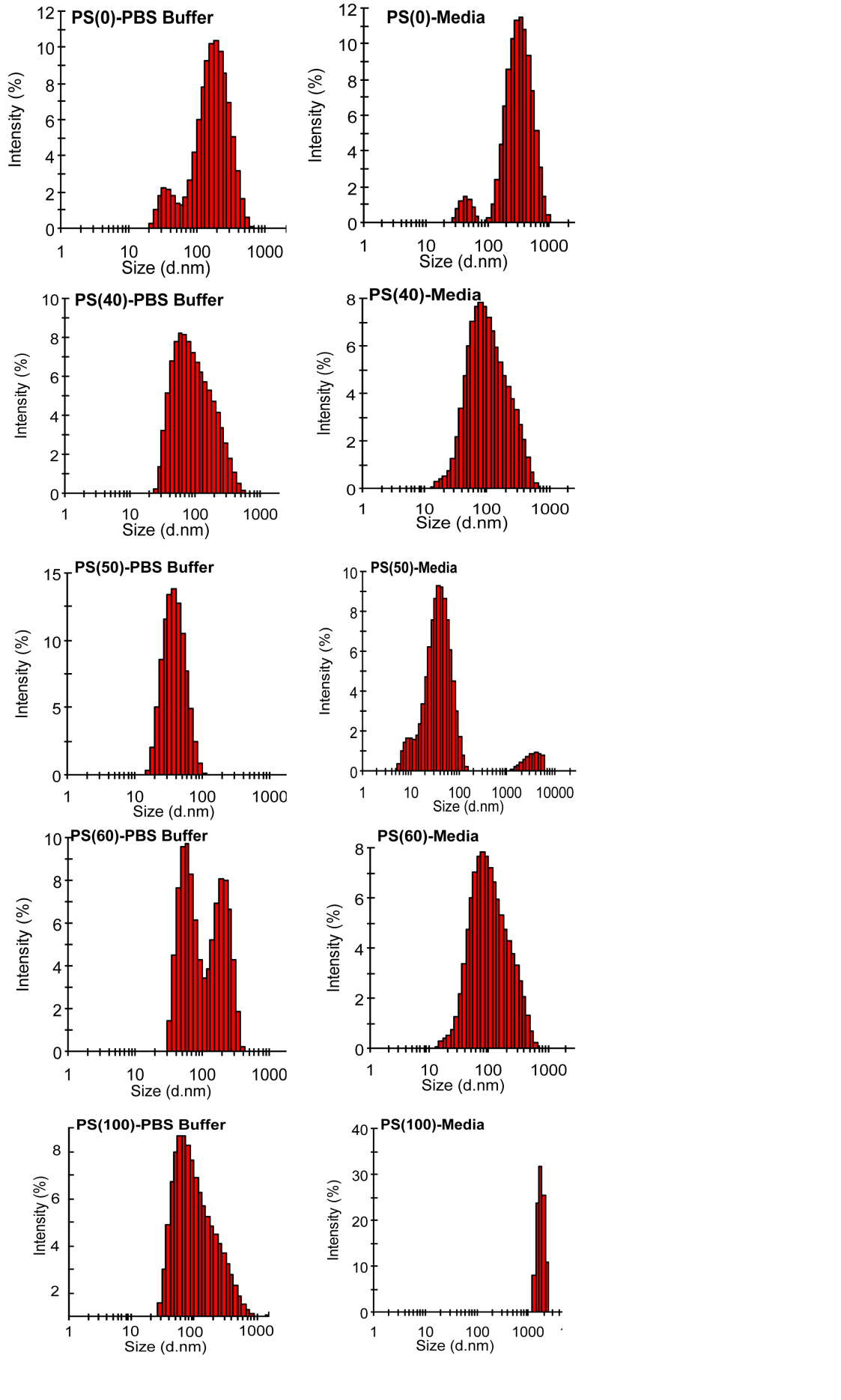


Fig. S4. Colloidal stability of PS-QD micelles in 10% fetal bovine serum. Size distribution analysis based on intensity distribution is shown. The PS-QD micelles with varying PL-PEG:PS mole ratio were incubated for 24 h in cell culture medium containing 10% fetal bovine serum and their size distribution were measured by a Zeta Nanosizer ZS. (A) PS (0) showed a shift in size after incubation in FBS. Peak shifted from 200 ± 94.9 nm nm to 300 ± 100.7 nm, (B) PS (40) were 125.4 ± 83.8 nm, (C) PS (50) showed two peaks at 40.5 ± 15.5 nm (86% intensity) and 2-3 μm (6% of total), (D) PS (60) micelles were 100 ± 62.4 nm, (E) PS (100) were 1 ± 2 μm respectively. (Three replicates were of each sample was analyzed by DLS with three runs per sample.


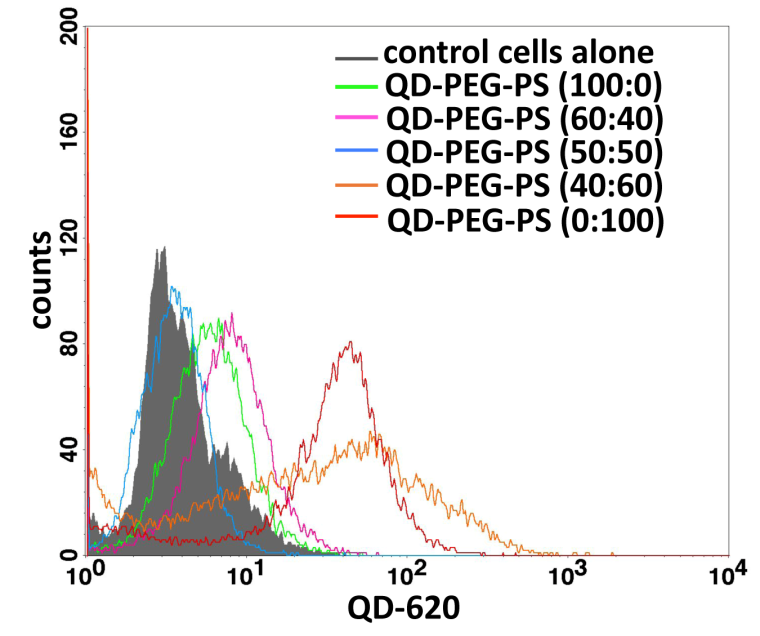


Fig. S5. Flow cytometry histograms of PS-QD micelle (25 pM) (with varying PEG:PS mole ratio, inset legend) uptake in J774A.1 monocytic cell line after 4 h incubation.


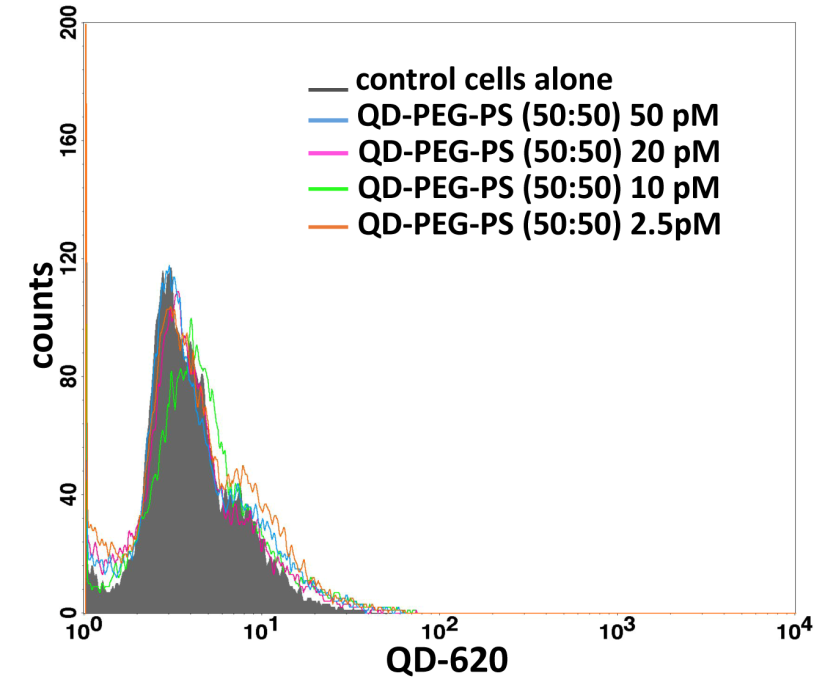


Fig. S6. Flow cytometry histograms of PS-QD micelle (50:50) uptake in J774A.1 monocytic cell line after 4 h incubation at 50, 20, 10 and 2.5pM concentrations (inset legend).


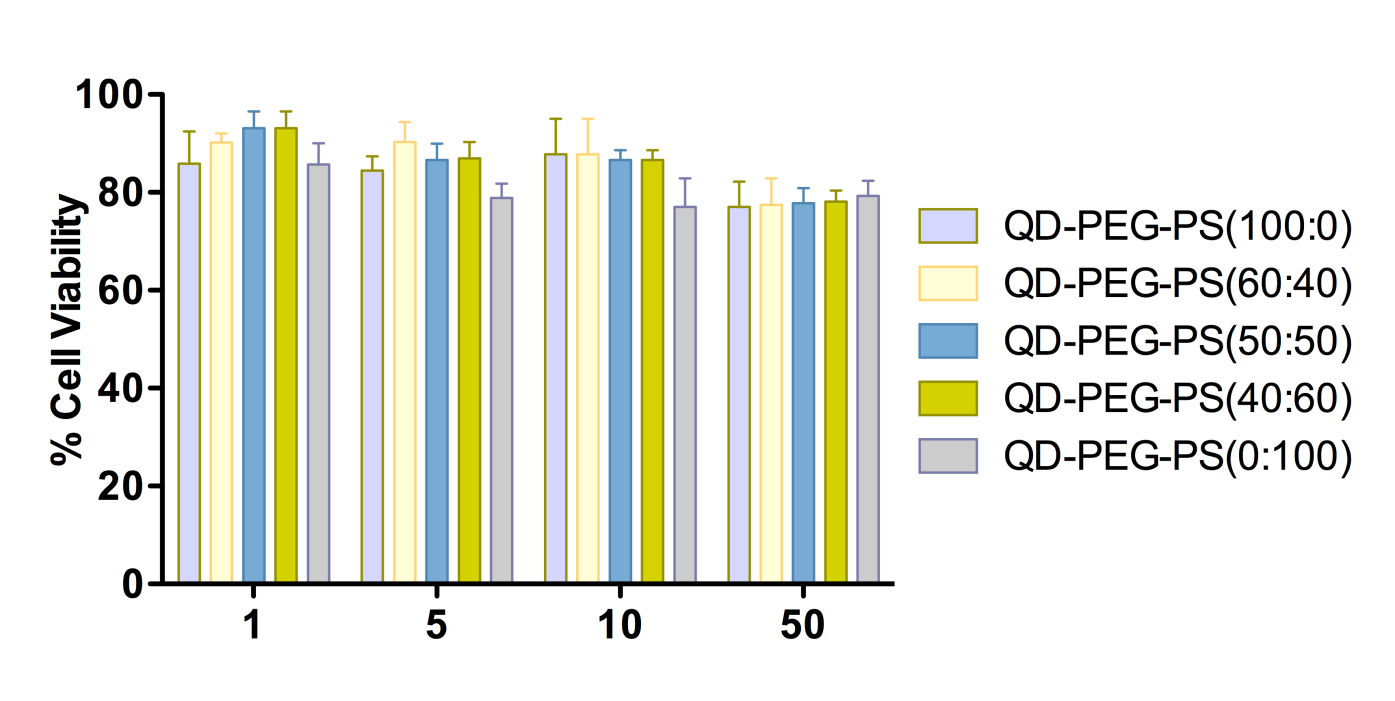


Fig. S7. The viability of J774A.1 cells as assessed by the MTT assay after incubating with various PS-QD micelles at 1, 5, 10 and 50 nM concentrations for 24 h.
